# Structures of FolT in substrate-bound and substrate-released conformations reveal a gating mechanism for ECF transporters

**DOI:** 10.1038/ncomms8661

**Published:** 2015-07-22

**Authors:** Qin Zhao, Chengcheng Wang, Chengyuan Wang, Hui Guo, Zhihao Bao, Minhua Zhang, Peng Zhang

**Affiliations:** 1 National Key Laboratory of Plant Molecular Genetics, Institute of Plant Physiology and Ecology, Shanghai Institutes for Biological Sciences, Chinese Academy of Sciences, Shanghai 200032, China

## Abstract

Energy-coupling factor (ECF) transporters are a new family of ABC transporters that consist of four subunits, two cytoplasmic ATPases EcfA and EcfA' and two transmembrane proteins namely EcfS for substrate-specific binding and EcfT for energy coupling. Here, we report the 3.2-Å resolution crystal structure of the EcfS protein of a folate ECF transporter from *Enterococcus faecalis*-*Ef*FolT, a close homologue of FolT from *Lactobacillus brevis*-*Lb*FolT. Structural and biochemical analyses reveal the residues constituting the folate-binding pocket and determining the substrate-binding specificity. Structural comparison of the folate-bound *Ef*FolT with the folate-free *Lb*FolT contained in the holotransporter complex discloses significant conformational change at the L1 loop, and reveals a gating mechanism of ECF transporters in which the L1 loop of EcfS acts as a gate in the substrate binding and release.

## Abstract (web summary)

Substrate specificity of ECF transporters is determined by the transmembrane EcfS protein subunit. Here Zhao *et al.* present substrate-bound and substrate-released structures of a bacterial folate transporter EcfS and suggest a gating mechanism for ECF transporters.

## Graphical abstract

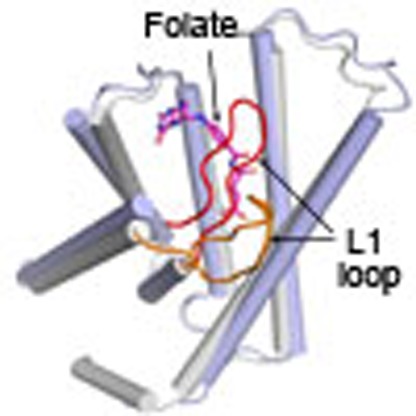

Energy-coupling factor (ECF) transporters were first discovered in bacteria in 1970s^1^; however, the molecular identities of this new family of ATP-binding cassette (ABC) transporters were not known until recent years^2^,^3^,^4^,^5^. Harnessing the energy of ATP hydrolysis, ECF transporters are responsible for micronutrient uptake from the environment^6^. ECF transporters are composed of four subunits, two cytoplasmic ATPases EcfA and EcfA', and two transmembrane proteins namely EcfS for substrate recognition and EcfT for energy coupling^7^,^8^,^9^. The most striking divergence, setting ECF transporters apart from the canonical ABC importers, lies in the substrate-binding proteins; the former utilize integral membrane proteins (EcfSs) for substrate binding, while the latter employ periplasmic solute-binding proteins to capture substrate^10^,^11^,^12^,^13^. The EcfT, EcfA and EcfA' proteins are termed the ECF module. Based on the features of ECF module, two groups of ECF transporters have been distinguished; in group I, each EcfS associates with a dedicated ECF module, while in group II, up to 12 EcfSs could share a common set of ECF modules^5^.

Because of the unique features of ECF transporters, a number of studies have been carried out to elucidate the underlying molecular basis of substrate specificity and transport mechanism. Structures of four different EcfS proteins in substrate-bound form have been solved, which include the riboflavin-specific RibU from *Staphylococcus aureus*, the thiamin-specific ThiT and biotin-specific BioY from *Lactococcus lactis*, and the nickel-specific NikM2 from *Thermoanaerobacter tengcongensis*^14^,^15^,^16^,^17^. These structures addressed the substrate-binding specificity of ECF transporters. Later, the structures of folate and hydroxymethylpyrimidine (the substrate specificity of this transporter is probably related to pyridoxine, and to be consistent with the structural reports, we use hydroxymethylpyrimidine here) transporters provided an overview of the architecture of the ECF transporter complex^18^,^19^. These two structures captured both in inward-facing substrate-free or inward-open conformation, presented a first snapshot towards elucidating the scenarios of the transport process and gave rise to the idea that the EcfS component rotates across the membrane to release the substrate into the cytoplasm. In particular, the structural elements involved in energy coupling between EcfT-EcfS and EcfA-EcfA' were disclosed, which are quite different from those of canonical ABC transporters. Recently, the structural basis of ECF module sharing among different EcfS proteins of group II ECF transporters was proposed by comparing the structures of pantothenate, folate and hydroxymethylpyrimidine ECF transporters from *Lactobacillus brevis* implemented with mutational analyses–different EcfS proteins use a common hydrophobic interaction surface composed of transmembrane helices 1, 2 and 6 to interact with the same EcfT or ECF module^20^. These structural observations are consistent with the results of functional analyses, and have further been used to explain events that occur during the transport process of ECF transporters.

Although a number of structures of individual substrate-binding proteins and holotransporter complexes are available, the mechanism of the large group of ECF transporters has not been elucidated. Of the currently available structures, four individual S-component structures are substrate-bound, and another three different S-component structures contained in the holotransporters are substrate-free. However, structures of a single S component crystallized in both states are lacking. Here we report the structure of the EcfS protein of a folate ECF transporter from *Enterococcus faecalis*-*Ef*FolT in complex with its substrate folate. Structural and biochemical analyses reveal the residues responsible for folate-specific binding. More importantly, structural comparison of the folate-bound *Ef*FolT with the previously reported structure of folate-free FolT from *L. brevis*-*Lb*FolT contained in the holotransporter complex reveals a gating mechanism of ECF transporters.

## Results

### Structure determination of substrate-bound *Ef*FolT

To study the underlying mechanism of substrate binding and release from ECF transporters, we focused on the crystallization of the EcfS proteins of folate ECF transporters-FolT. Several FolT proteins from different species were homogenously purified, but only the FolT protein from *E. faecalis* (*Ef*FolT) could be successfully crystallized. Numerous efforts were made to optimize the crystals, including the addition of 10-fold excess (molar ratio) amount of folate in the purified protein solution, which was found to be essential for the growth of diffractable crystals. In the end, an anisotropic X-ray data set (2.7–3.5 Å) was collected by screening hundreds of crystals. The data were truncated to 3.2-Å resolution based on the analyses of the UCLA-MBI diffraction anisotropy server^21^. The structure of *Ef*FolT was determined with molecular replacement method using the substrate-free *Lb*FolT (*Lb*, *L. brevis*) structure as a template. There are six *Ef*FolT molecules in one asymmetric unit. The electron density for molecules A, B, C and D are quite good for modelling, while the density for molecules E and F are poor and the models are built based on molecule A structure (Supplementary Fig. 1). The statistics of data collection and structure refinement are summarized in Supplementary Table 1.

**Figure 1:**
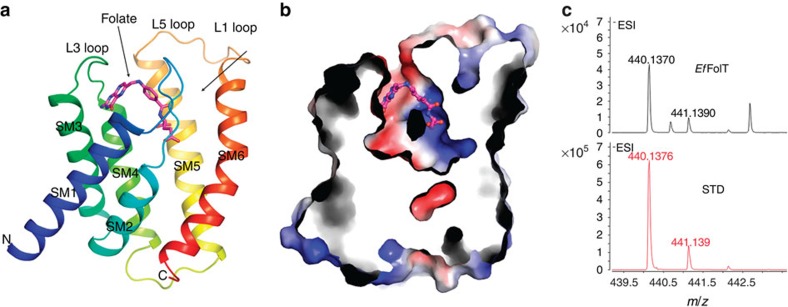
Overall structure of *Ef*FolT. (**a**) Overall structure of *Ef*FolT in ribbon cartoon. The six transmembrane helices 1–6 (SM1-6) are coloured from blue to red. The bound substrate folate is shown with a stick model coloured in magenta. (**b**) A cross-section drawing of *Ef*FolT shows the folate-binding pocket (blue and red colours represent positive and negative charges, respectively). (**c**) Mass spectrometry results of the substrate bound with *Ef*FolT. Lower panel shows the folate standard; upper panel shows the substrate bound with *Ef*FolT.

**Figure 2:**
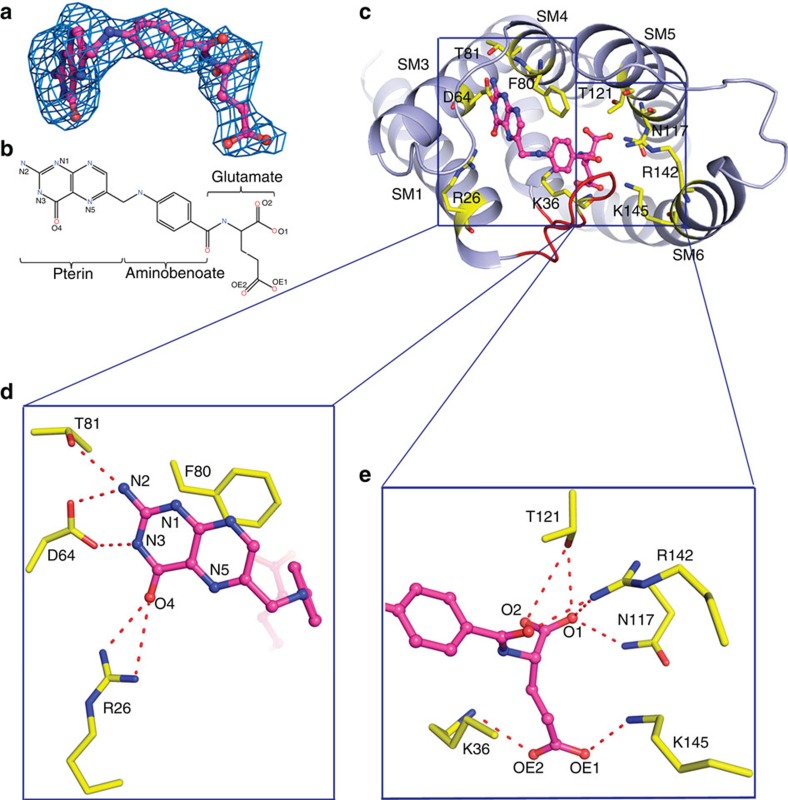
Folate-binding site of *Ef*FolT. (**a**) Electron density of folate at the substrate-binding pocket of *Ef*FolT (Fo-Fc density contoured at 2.0 σ level in molecule A). Folate is shown with a ball-and-stick model in magenta. (**b**) Chemical structure of folate. (**c**) Top view of the folate-binding pocket of *Ef*FolT. Structure of *Ef*FolT is shown with light-blue ribbons, and residues that line the pocket are shown with yellow sticks. (**d**, **e**) Close-up views of the interactions of pterin moiety (**d**), and glutamate and aminobenoate moieties (**e**) of folate with surrounding residues. The hydrogen bonds are indicated by dashed lines.

There are small conformational differences among the six molecules (root mean square deviations/RMSD of 0.7–1.2 Å). The overall structure of *Ef*FolT is similar to the reported structures of EcfS proteins (RMSD of 1.5–3.3 Å) which consist of six transmembrane helices (SM1-6, transmembrane helices 1–6 of EcfS) that form a helix bundle. The N-terminus and C-terminus are both at the cytoplasmic side. Three loops L1, L3, L5 connect SM1-SM2, SM3-SM4 and SM5-SM6, respectively, from the periplasmic side (Fig. 1a). A pocket with the depth of 24 Å and volume of 1,230 Å^3^ is formed by the six transmembrane helices near the periplasmic side and is covered by the L1 loop from top (Fig. 1b). The substrate bound in the pocket is verified by mass spectrometry to be folate (Fig. 1c) and further confirmed by the electron density map in the pocket (Fig. 2a).

### The folate-binding site

**Figure 3:**
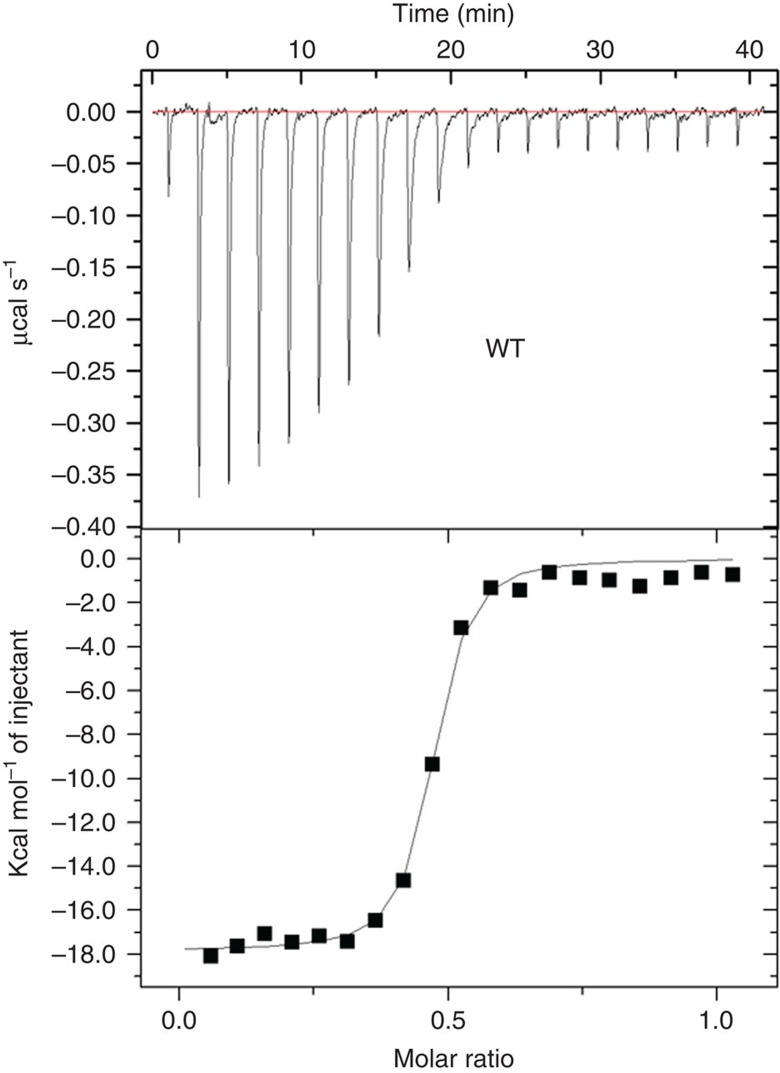
Folate-binding affinity of *Ef*FolT assayed by isothermal titration calorimetry. The titration curve of folate binding to wild-type *Ef*FolT is presented.

The folate molecule adopts an ‘L'-shaped conformation, with the pterin moiety forming the short arm and the aminobenzoate and glutamate moieties forming the long arm (Fig. 1a,b). A similar conformation of folate has been found in the human folate receptor FR^22^. The recognition of folate is mainly through the pterin and glutamate moieties by a number of residues protruding from SM1, L1 loop and SM3-6 (Fig. 2b,c). Specifically, the pterin ring of folate forms π–π interactions with the parallel side chain of Phe80. The pterin N2 atom forms two hydrogen bonds with the side chains of Asp64 and Thr81, the N3 atom forms a hydrogen bond with the carboxyl oxygen of Asp64, and O4 atom forms two hydrogen bonds with the guanidine group of Arg26 (Fig. 2d). The glutamate moiety of folate is stabilized mainly through six hydrogen bonds. Specifically, the O1 and O2 atoms of glutamate group form four hydrogen bonds with the side chains of residues Asn117, Thr121 and Arg142; and the OE1 and OE2 atoms form two hydrogen bonds with the side chains of Lys36 and Lys145. In addition, Arg142 forms another hydrogen-bonding interaction with the carbonyl oxygen of aminobenzoate moiety (Fig. 2e). The extensive interactions between folate and *Ef*FolT are consistent with the high binding affinity determined by Isothermal Titration Calorimetry (ITC) experiments (dissociation constant/*K*_D_ of 29.8 nM for folate) (Fig. 3), and the previously reported high binding affinity of *Lc*FolT (*Lc*, *L. casei*) with folate^23^. In addition, the above residues involving folate binding are highly conserved, suggesting that a common folate recognition mode could exist among the FolT proteins of ECF transporters (Supplementary Fig. 2).

To validate the structure observations, we mutated the residues interacting with folate and tested their effects on substrate binding using ITC (Table 1 and Supplementary Figs 3 and 4). The results show that the replacement of Phe80 with Ala can abolish folate binding, suggesting the essential role of Phe80 in folate stabilization. Nevertheless, the F80W mutant still retains considerable binding of folate (*K*_D_=499 nM). In this case, the large side chain of Trp may lead to some confliction with the pterin ring but still retain partial stacking interaction. Among the residues interacting with the pterin head via hydrogen bonds, mutation of Arg26 or Thr81 to Ala decreases the folate-binding affinity by a factor of 8 or 27; while replacement of Asp64 with Ala abolishes the folate binding. When breaking the hydrogen-bonding interactions on the glutamate side, the substrate-binding affinity of N117A mutant is slightly decreased, and that of T121A and K145A/T mutants is decreased by 10-fold and 110-fold respectively, whereas the folate-binding affinity of R142A or K145D mutant is destroyed. For the residue Lys36 from the L1 loop, it is conserved but is replaced with Arg in some species (Supplementary Fig. 2). Consistently, the folate-binding affinity of K36R mutant is slightly affected, and that of K36A mutant is significantly reduced by a factor of 180 while K36D mutant loses the folate-binding affinity, which suggests that the hydrogen bond formed between the residue Lys/Arg36 from the L1 loop and the carboxyl group of folate is critical for the folate binding (Table 1). Taken together, the contributions of these residues to folate binding can be ranked according to the above results: Asp64; Arg142; Phe80> Lys36; Lys145> Thr81; Arg26; and Thr121>Asn117.

### Conformational change of FolT induced by folate binding

**Figure 4:**
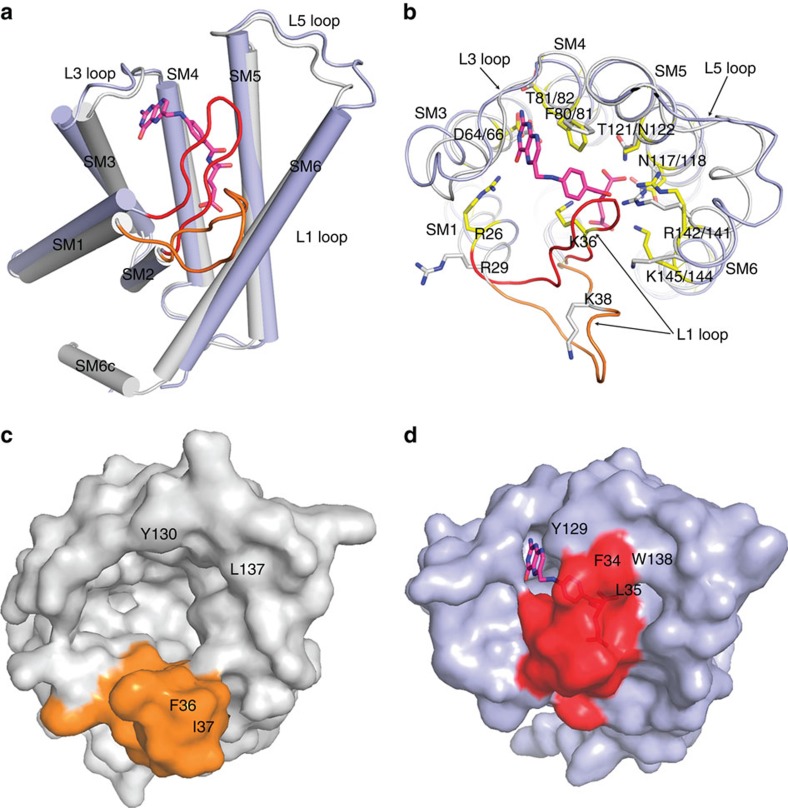
Conformational differences of FolT in substrate-bound and substrate-free states. (**a**) Superimposition of the structures of substrate-bound *Ef*FolT (light-blue cylinders, *Ef*FolT molecule A structure is used) with substrate-free *Lb*FolT (grey cylinders) shows the overall conformational difference. The L1 loops of *Ef*FolT and *Lb*FolT are coloured in red and orange, respectively; the folate binding with *Ef*FolT is shown with a magenta stick model. (**b**) Top view of (**a**) shows the conformational differences of the residues constituting the folate-binding pocket. Residues comprising the folate-binding pocket of *Ef*FolT and *Lb*FolT are shown with yellow and grey sticks, respectively. (**c**, **d**) Surface model show the folate-binding pocket (top view) of *Lb*FolT (**c**) and *Ef*FolT (**d**). Colour codes are same as in (**a**).

The two EcfS proteins of folate ECF transporters, *Ef*FolT and *Lb*FolT share sequence similarity of 54.9% and identity of 33.0%, and the structure of *Ef*FolT represents the folate-binding state conformation, while the structure of *Lb*FolT contained in the folate ECF transporter complex represents the folate-released state conformation; therefore, we compare these two structures to investigate the underlying mechanism of substrate binding and release. Superimposition of the *Ef*FolT and *Lb*FolT structures reveals a RMSD of 1.5 Å, and significant conformational difference is observed at the L1 loop. In *Ef*FolT, the L1 loop covers on the substrate-binding pocket and adopts a ‘closed' conformation, while in *Lb*FolT, the same loop flips away from the pocket and adopts an ‘open' conformation (Fig. 4a,b). Based on this data, we suggest that folate binding can induce a conformational change at the L1 loop. In the folate-released or free state, the L1 loop adopts an ‘open' conformation to leave the pocket widely open as represented by the *Lb*FolT structure (Fig. 4c); once folate binds into the pocket, residue Arg26 from the C terminal of SM1, and residue Lys36 from the L1 loop form three hydrogen bonds with folate, which we propose pulls the L1 loop from the ‘open' to the ‘closed' conformation as represented by the *Ef*FolT structure (Fig. 4b,d). In addition, residues (i.e., residues Phe34 and Leu35) from the L1 loop stack against the residues constituting the L5 loop (i.e., residues Leu125, Tyr129 and Trp138), which may stabilize the ‘closed' conformation of L1 loop in *Ef*FolT (Fig. 4d). Mutation of these residues can decrease folate binding (Table 1 and Supplementary Fig. 5). These observations suggest that the L1 loop acts as a gate in controlling the substrate binding and release of the folate ECF transporter.

## Discussion

In this work, the structure of the EcfS protein of a folate ECF transporter-*Ef*FolT-is determined in the folate-bound conformation which revealed key residues responsible for substrate-specific binding. For the first time, the substrate-binding-induced conformational changes of the EcfS protein of ECF transporters can be clearly seen by comparing the structures of substrate-bound *Ef*FolT with substrate-free *Lb*FolT in the folate ECF transporter complex captured in inward-open conformation. Based on the data presented here, we suggest that the L1 loop acts as a gate in folate binding and release and that folate binding induces the conformation of the gate to change from ‘open' to ‘closed'. Our data is supported by a previous report suggesting that the L1 loop of ThiT undergoes conformational changes upon thiamine binding^24^. These results raise another important question during the transport process: what drives the change of the L1 loop from the ‘closed' conformation to the ‘open' conformation to release the substrate? To provide insights of this question, we modelled the folate-bound *Ef*FolT structure to the quarternary folate ECF transporter complex-*Lb*ECF-FolT captured in inward-open state, and extensive interactions are observed between the transmembrane helices 1, 2, 6 of *Ef*FolT and coupling helices 2/3 of EcfT protein, which are commonly observed among group II ECF transporters^20^. In addition, clashes are observed between the L1 loop of *Ef*FolT and the transmembrane helix 3 of *Lb*EcfT (Supplementary Fig. 6). These observations indicate that the driving force is closely related with EcfT. Here, we postulate that the force(s) driving the conformational change of L1 loop come from the relative movement of EcfS against EcfT during the transport process (Supplementary Fig. 7): energy coupling helices 2/3 (CH2/3) and probably transmembrane helices of EcfT undergo conformational changes following the ATP hydrolysis in EcfA/A' proteins, which may further lead to the changes of transmembrane helices of EcfS, especially SM1 and SM6. As small conformational changes in SM1, SM2, SM6 or L1 loop can affect the residues essential for substrate binding (Figs 2 and 3), i.e., Arg26 from SM1, Lys36 from L1 loop and Arg142 and Lys145 from SM6 in *Ef*FolT, the interaction of folate with these residues, especially residues Arg26 and Lys36 which adopt different conformations in *Lb*FolT and *Ef*FolT could initially be broken. As a result, breaking of these interactions will greatly lower the binding affinity of folate with FolT and speed up the conformational change of L1 loop from ‘closed' to ‘open', which finally leads to substrate release (evidenced by the fact that the substrate folate cannot bind with the inward-open folate ECF transporter complex-*Lb*Ecf-FolT-as detected by ITC (Supplementary Fig. 5)). Similar mechanism might exist among other ECF transporters as there are essential residues protruding from the above mentioned structural elements and involving substrate binding. Taken together, our results and analyses suggest a gating mechanism of ECF transporters, in which the L1 loop acts as a gate in substrate binding and release.

We also note a recent study about the gating mechanism of RibU, in which the authors performed molecular dynamic simulation analyses and suggested that the L5 loop could serve as a gate in riboflavin binding and release^25^. However, our structural data of the folate ECF transporter do not support this conclusion. Although we also observed conformational change at the L5 loop, we considered it as the stabilization factor of the L1 loop. To be more critical, as the L5 loop of EcfS is distant from the energy-coupling scaffold EcfT in the transporter complex, if L5 loop acts as a gate, then the driving force of the conformational change is questionable. Nevertheless, we cannot exclude the possibility that ECF transporters use different gating mechanisms for substrate binding and release, which will require further structural analysis to determine.

## Methods

### Gene cloning and protein purification

The gene encoding the S component of the folate ECF transporter from *E. faecalis, EfFolT* was cloned into pET28a vector using NdeI and XhoI (primers used are: for-AAAAACATATGATGACAAAGAAAAAATTTGG, and rev- AAAAACTCGAGTTATTGATCTAATTCAGATA). A tag of six histidine residues was added at the N-terminus of the *Ef*FolT. The plasmid was transformed into *Escherichia coli* BL21 (DE3) and induced by 0.25 mM β-D-thiogalactopyranoside (IPTG) at *A*_600_ of about 1.2. After 14 h at 37 °C, the cells were harvested, and homogenized in buffer A (100 mM NaCl, 20 mM Tris-HCl pH 8.0), and lysed using a French press. Cell debris was removed by centrifugation. The supernatant was collected and applied to ultracentrifugation at 150,000*g* for 1 h. Membrane fraction was incubated with 2% (w/v) *n*-nonyl-β-D-glucopyranoside (NG; Anatrace) for 2 h at 4 °C. After another centrifugation step at 20,000*g* for 45 min, the supernatant was loaded onto a Ni^2+^-NTA affinity column (Qiagen), and washed with buffer B (100 mM NaCl, 20 mM Tris-HCl pH 8.0 and 0.4% NG) plus 25 mM imidazole. The protein was eluted from the column using buffer B plus 250 mM imidazole, and was concentrated to around 10 mg ml^−1^ before further purified by gel filtration (Superdex-200, GE Healthcare) in buffer B. The peak fraction was collected and concentrated to ∼5 mg ml^−1^ for crystallization.

### Identification of substrate bound with *Ef*FolT using liquid chromatography–mass spectrometry

Equivalent volume of acetonitrile was added to the purified *Ef*FolT solution (10 mg ml^−1^, 500 μl) to denature the protein. Then the sample was sonicated for15 min at room temperature to release the substrate. After centrifugation at 12,000 r.p.m. for 10 min, the supernatant was filtered through a 0.22-μm membrane before loaded onto the C18 column connected with Agilent G6520A accurate-mass quadrupole time-of-flight liquid chromatography–mass spectrometry system.

### Crystallization and structure determination

For crystallization of *Ef*FolT, 10-fold molar excess amount of folate was added to the purified protein solution before crystallization. The crystallization was performed at 20 °C using sitting drop vapour diffusion method. Small crystals were found in several conditions. After extensive optimization, diffraction-quality crystals were obtained under the condition containing 15% (w/v) polyethylene glycol 2000, 0.5 M NaCl and 0.1 M sodium dihydrogen phosphate, pH 6.8. Crystals used for data collection were flash-frozen in liquid nitrogen. All data sets were collected at the Shanghai Synchrotron Radiation Facility and processed with HKL2000. The crystals belong to the space group P3_1_ with unit cell dimensions of *a*=92.8 Å, *b*=92.8 Å, *c*=183.4 Å. The diffraction data were checked by the UCLA-MBI diffraction anisotropy server to be anisotropic (2.7–3.5 Å) and truncated to 3.2 Å resolution (*Ef*FolT-truncate).

To solve the structure, molecular replacement was used with the structure of FolT of *Lb*FolT-ECF transporter as a template (PDB ID: 4HUQ). Six molecules were found in one asymmetric unit, and four of them (molecules A, B, C, D) have high-quality electron density which allowed the auto-building of the Cα trace with PHENIX^26^. The building of the remaining residues was carried out manually in COOT^27^. The molecules E and F have poor electron density which may be because of a crystallographic defect, and the model building was based on molecule A structure. The final model was refined using PHENIX (*R*_work_/*R*_free_ =0.293/0.356). The Ramachandran statistics are 93.0% for favoured region and 7.0% for allowed region. The statistics of data collection and refinement are summarized in Supplementary Table 1.

### Isothermal titration calorimetry analysis

ITC experiments were performed with a MicroCal ITC200 system (Malvern) at 20 °C in a buffer containing 20 mM Tris-HCl, pH 8.0, 100 mM NaCl, 0.018% (w/v) *n*-Dodecyl-β-D-Maltopyranoside. The syringe was filled with folate (Sigma, Cat: F7876) and the sample cell was filled with *Ef*FolT protein. The concentrations of folate and protein were optimized for all experiments. The folate was added to the protein by sequential injections of 2-μl aliquots followed by 120 s of equilibration after each injection and there were 20 injections in total. For analysis, the heat released by each injection was integrated, and the background was subtracted. The data were fit to the Wiseman isotherm with the Origin ITC analysis package. The experiments were repeated at least twice for each sample.

## Additional information

**Accession codes:** Structural data have been deposited in the Protein Data Bank under accession code 4Z7F.

**How to cite this article:** Zhao, Q. *et al.* Structures of FolT in substrate-bound and substrate-released conformations reveal a gating mechanism for ECF transporters. *Nat. Commun.* 6:7661 doi: 10.1038/ncomms8661 (2015).

## Supplementary Material

Supplementary Information Supplementary Figures 1-7 and Supplementary Table 1

## Figures and Tables

**Table 1:** Folate-binding affinity of *Ef*FolT substrate-binding pocket mutants assayed by isothermal titration calorimetry.

**Mutants**	**Dissociation constant/*****K***_**D**_ **(nM)**
WT	29.8±4.7
R26A	251.9±22.0
D64A	No binding*
T81A	813.5±162.1
F80A	No binding
F80W	498.8±25.8
N117A	68.6±6.4
T121A	288.1±21.1
R142A	No binding
K145A	3301±198
K145T	3,690±222
K145D	No binding
K36A	5,403±549
K36R	91.5±7.5
K36D	No binding
F34A	68.4±4.7
Y129A	634.7±33.6
W138A	147.3±15.0

Binding affinity of folate to *Ef*FolT wild type and mutants is given.

^*^=undetectable with ITC. ±indicates systematic errors calculated by the program ‘origin'.
