# Supplementary material for: Structures of FolT in substrate-bound and substrate-released conformations reveal a gating mechanism for ECF transporters
Source: Nat Commun. 2015 Jul 22;6:7661. doi: 10.1038/ncomms8661 (PMC4525288; doi:10.1038/ncomms8661)
Supplement: Supplementary Information — Supplementary Figures 1-7 and Supplementary Table 1 [file ncomms8661-s1.pdf]

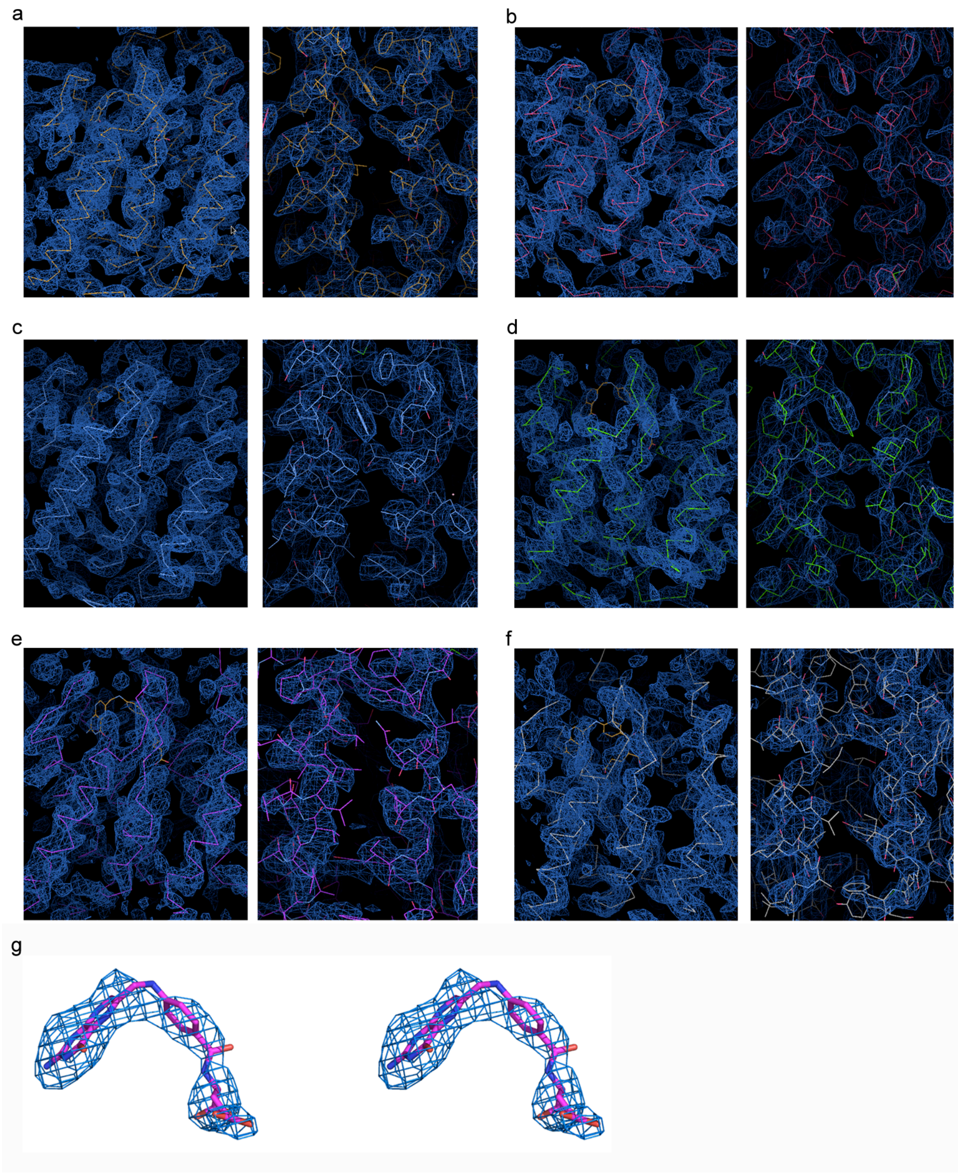

Supplementary figure 1. Electron density map of *Ef*FolT. a-f, 2FoFc electron densities of molecules A (yellow), B (red), C (blue), D (green), E (magenta), and F (gray). The left panels show the densities for the whole molecules (Cαs are shown) while the right panels show the densities for transmembrane helices 4 and 5 (side chains are shown). The densities for molecules A-D and E-F are contoured at 1.5 σ and 1.0 σ, respectively. **g**, Stereo view of the electron density of folate at the substrate binding pocket of *Ef*FolT (Fo-Fc density contoured at 2.0 σ level in molecule A)**.**

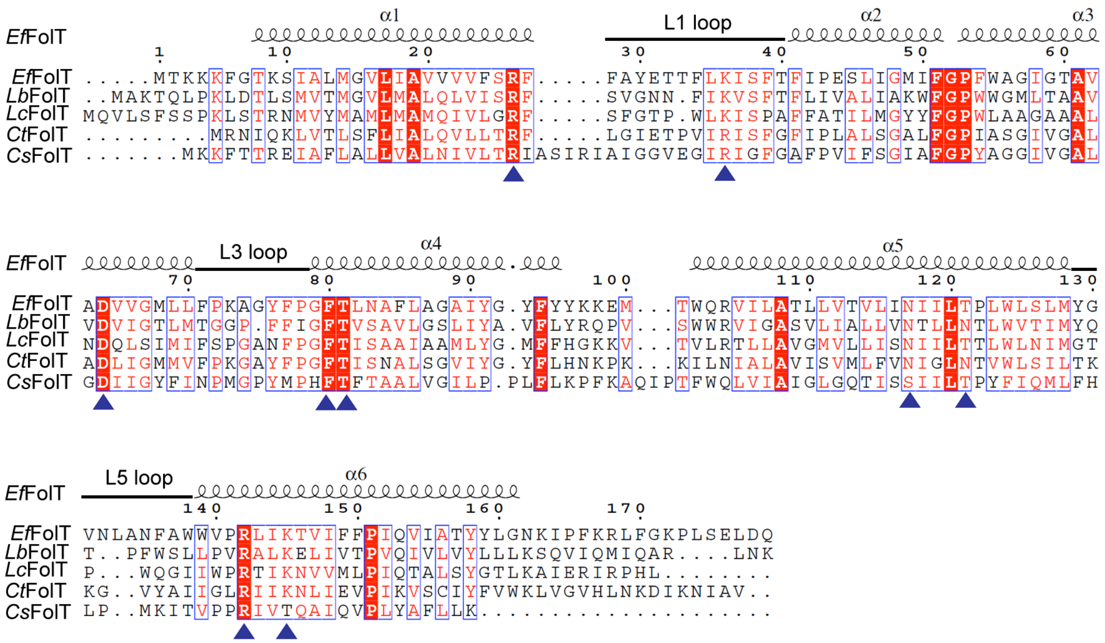

Supplementary figure 2. Sequence alignment of FolT from representative species. The amino acid sequences of FolT homologues from 5 bacterial species are aligned, with the secondary structural elements of *Ef*FolT indicated above the sequences. Invariant residues are highlighted in red whereas conserved amino acids are boxed. Residues forming hydrogen bonding and hydrophobic interactions with folate are indicated with blue triangles. The FolT homologs are from *Enterococcus faecalis* (*Ef*, WP_002385065), *Lactobacillus brevis* (*Lb*, WP_01166775)*, Lactobacillus casei group* (*Lc*, WP_003567081), *Clostridium tetani* (*Ct*, WP_011099884), *Caldanaerobacter subterraneus* (*Cs*, WP_011026453).

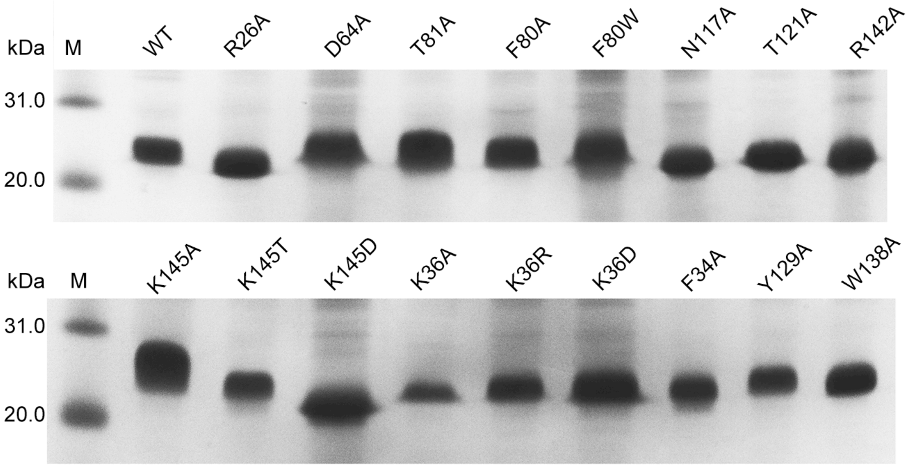

Supplementary figure 3. SDS-PAGE shows the purification results of *Ef*FolT wild type and mutants. The gels are stained with commassie blue. The purified proteins are further used for ITC experiments.

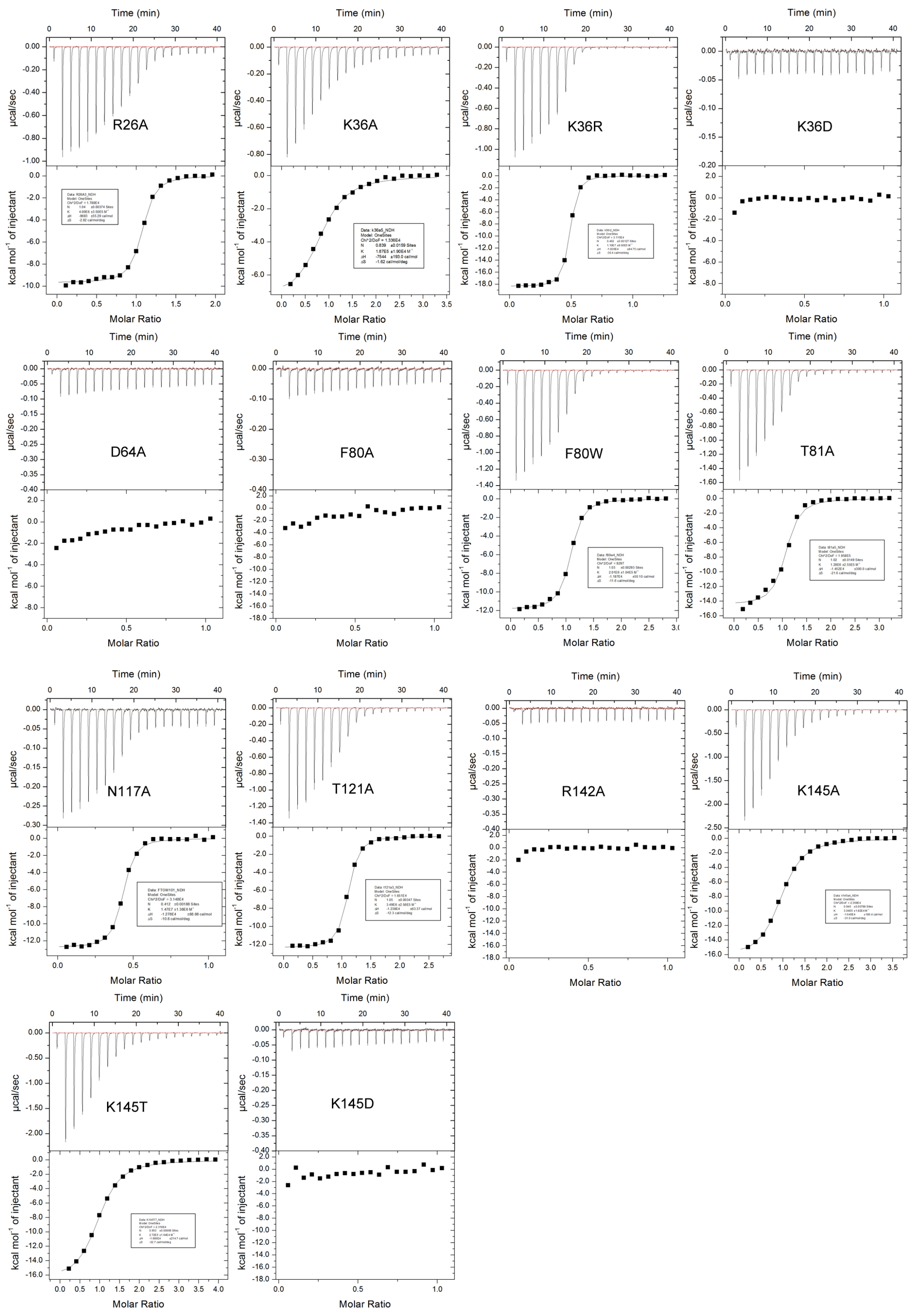

Supplementary figure 4. The titration curves of folate to *Ef*FolT substrate binding pocket mutants.

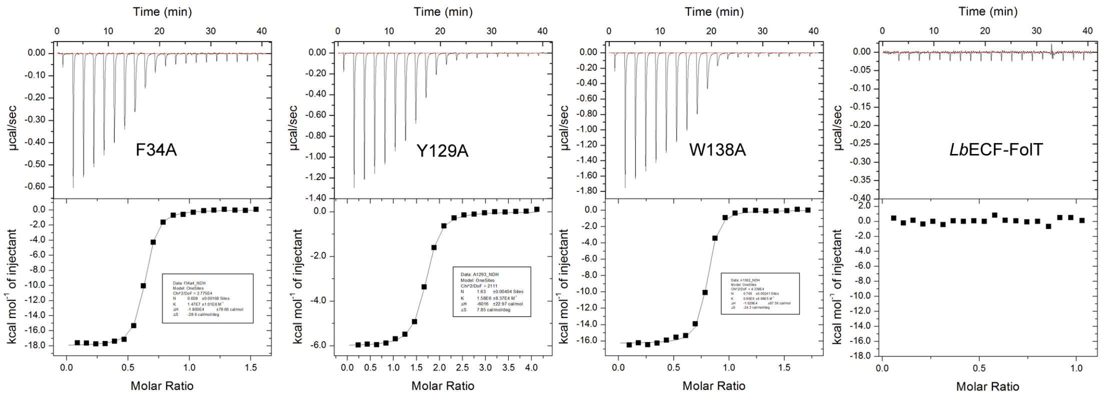

Supplementary figure 5. The titration curves of folate to *Ef*FolT L1/L5 loop mutants and *Lb*ECF-FolT complex.

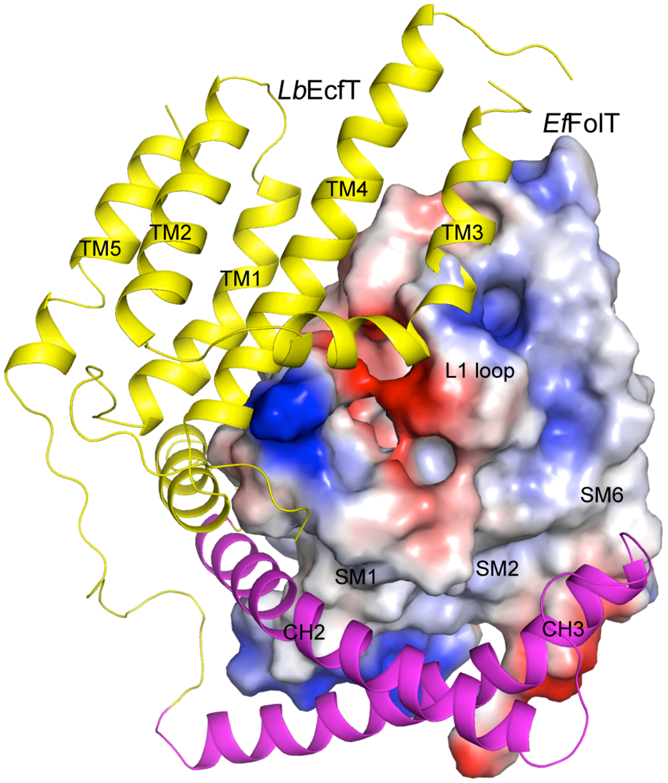

Supplementary figure 6. Structure modeling shows the interactions of folate-bound *Ef*FolT with *Lb*EcfT. *Ef*FolT is shown with electrostatic surface potential (blue and red colors represent positive and negative charges, respectively), *Lb*EcfT is shown in ribbon cartoon with transmembrane helices 1-5 (TM1-TM5) colored in yellow and coupling helices 2-3 (CH2-CH3) colored in magenta.

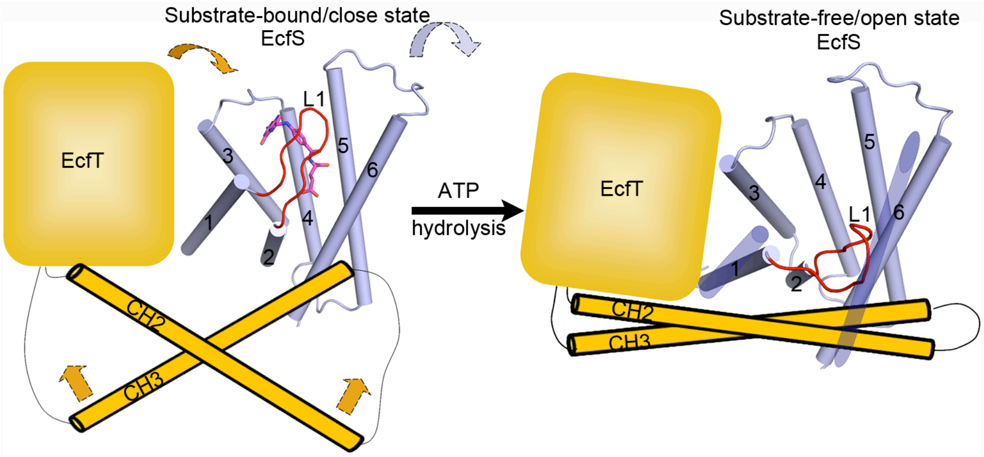

Supplementary figure 7. Assumed transition model from substrate-bound state to substrate-free state. Energy coupling helices 2/3 (CH2/3) and probably transmembrane helices of EcfT (shown with orange square) undergo conformational changes (indicated with orange arrows) following the ATP hydrolysis in EcfA/A' proteins, which may further lead to the conformational changes of the transmembrane helices of EcfS (indicated with blue arrows), especially transmembrane helices 1 and 6 (the possible conformational changes are indicated with shaded cylinders) forming interactions with CH2/3.These conformational changes will finally drive the L1 loop of EcfS from "close" to "open" to release the substrate. EcfT is shown with orange cartoon, and EcfS is shown with blue cylinders.

Supplementary table 1 Statistics of data collection and structure refinement

	*Ef*FolT	*Ef*FolT-truncate[a]
**Data collection**		
Space group	$P3_1$	
Cell dimensions		
a, *b*, *c* (Å)	92.8, 92.8, 183.4	
α, β, γ (º)	90, 90, 120	
Resolution (Å)	50.0-3.2 (3.29-3.20)[b]	
$R_{merge(\%)}$	0.138 (0.976)	0.117 (0.293)
$I/\sigma I$	6.85 (1.1)	7.87 (4.79)
Completeness (%)	98.1 (96.3)	76.7 (19.51)
Redundancy	3.5	3.4
**Refinement**		
Resolution (Å)		3.2
No. reflections		22431
R_{work}/R_{free} [c]		0.293/0.356
No. atoms		7898
Protein		7706
Ligand		192
B-factors (Å^2)		
Protein/L1 loop		Mol_A : 90.2/96.1
(29-39 aa)		Mol_B : 87.6/101.6
		Mol_C : 87.5/90.4
		Mol_D : 87.7/110.0
		Mol_E: 156.9/144.6
		Mol_F: 147.3/140.3
Folate		A: 125.1
		B: 141.2
		C: 119.6
		D: 125.0
		E: 130.7
		F: 163.3
R.m.s deviations		
Bond lengths (Å)		0.017
Bond angles (º)		2.43

[a] Data were truncated by the UCLA-MBI diffraction anisotropy server.

[b] Numbers in parentheses represent the highest-resolution shell.

One crystal was used for data collection.
